# Primary Validation of the Submandibular Skinfold as an Anthropometric Measurement of Cardiometabolic Risk in People with Intellectual Disabilities

**DOI:** 10.3390/ijerph20031658

**Published:** 2023-01-17

**Authors:** Paloma Ferrero-Hernández, Claudio Farías-Valenzuela, Gerson Ferrari, Sebastián Álvarez-Arangua, Hans Villalobos-Flores, Pedro Valdivia-Moral

**Affiliations:** 1 Facultad de Educación y Cultura, Universidad SEK, Santiago 7520318, Chile; 2 Instituto del Deporte, Universidad de las Américas, Santiago 9170022, Chile; 3 Facultad de Ciencias de la Actividad Física, el Deporte y la Salud, Universidad de Santiago de Chile (USACH), Santiago 9170022, Chile; 4 Facultad de Ciencias de la Salud, Universidad Autónoma de Chile, Santiago 7500912, Chile; 5 Exercise and Rehabilitation Sciences Institute, School of Physical Therapy, Faculty of Rehabilitation Science, Universidad Andres Bello, Santiago 7591538, Chile; 6 Functional Movement, Santiago 8320000, Chile; 7 Department of Didactics of Musical, Plastic and Body Expression, Faculty of Education, University of Granada, 18071 Granada, Spain

**Keywords:** intellectual disability, cardiometabolic risk, obesity, anthropometric measurements, BMI, submandibular skinfold

## Abstract

The accumulation of body fat is an important cardiometabolic risk factor; however, there is no consensus about which measure is more reliable for the assessment of cardiometabolic risk in people with intellectual disabilities. The aim of the present study was to primarily validate the submandibular skinfold as an anthropometric measurement of cardiometabolic risk in children, adolescents, and adults with intellectual disabilities, using a cross-sectional study made up of 131 people (67.2% men) with mild and moderate intellectual disability. The cardiometabolic risk indicators used were: body mass index (kg/m^2^), neck circumference (cm), waist circumference (cm), calf circumference (cm) and waist-to-height ratio. Moderate correlations were demonstrated between the submandibular skinfold measure and the anthropometric measurements analyzed in the three age categories, showing the highest correlation (r = 0.70) between the submandibular skinfold and BMI in the adolescent group and waist-to-height ratio in adults. The implementation of the submandibular skinfold measurement is suggested as an easy, fast, and minimally invasive anthropometric measurement as part of the physical and nutritional evaluation for the assessment of cardiometabolic risk in people with intellectual disabilities.

## 1. Introduction

The prevalence of obesity is a global problem that has increased in recent years, reaching pandemic levels, and this is associated in all its degrees with an increased risk of chronic diseases and mortality from all causes [1,2,3]. Evidence has shown associations between a high body mass index (BMI) and a significant increase in mortality from cardiovascular diseases, diabetes, and kidney disease [4]. The association between BMI and mortality is stronger at younger than at older ages, decreasing life expectancy from age 40 in obese people (BMI ≥ 30.0 kg/m^2^) compared to individuals of healthy weight [5]. The risk of multimorbidity and obesity is even higher in people with an intellectual disability (ID) compared to the general population [6], leading to poorer health than their peers without disabilities. In addition, people with ID are at an increased risk of cardiometabolic disorders due to high rates of chronic inflammation, high use of psychotropic drugs, and limited access to health care, in addition to modifiable factors such as sedentary lifestyle and unhealthy diets, which contribute to early morbidity and mortality in this population [7,8].

For the assessment of cardiometabolic risk, BMI has been widely considered as a simple and easy-to-calculate anthropometric metric to assess obesity and adverse metabolic outcomes in children, adolescents, and adults as it is well correlated with adiposity [9], which, as well as waist circumference, is similarly strongly associated with cardiovascular disease in young and older adults of both sexes [10]. However, it is not considered the most accurate indicator because people with a normal body weight and BMI can also present metabolic disorders [11]. Waist circumference, waist-to-height ratio and waist-to-hip ratio have also been considered as indicators of central obesity, appearing to be slightly better predictors than BMI [12,13] and complement the identification of cardiovascular risk factors. Skinfolds represent a more direct and easy-to-use measure of adiposity for examining trunk and overall obesity, which may give a better insight of the relationship between adiposity, cardio-metabolic risk, and later obesity in children and adolescents [14], suggesting that skinfold thickness could be used to measure obesity with the advantage of indicating fat distribution [15]. BMI has been validated as a stronger indicator than skinfold thickness in overweight youth [16]; however, the prediction of cardiometabolic risk is more optimal when both measurements are combined. Neck and calf circumferences have been negatively and independently associated with the risk of cardiovascular disease at 10 years, contributing to the prediction of cardiovascular risk beyond traditional anthropometric measurements [17,18].

In people with an ID, BMI cannot always be used due to neuromuscular coordination disorders that could impair the ability to step on a scale, as well as psychological barriers that can make conventional body-weight measurement difficult. However, BMI can be recommended for the estimation of cardiometabolic risk complemented with the body adiposity index [19]. In this context, the submandibular skinfold has been proposed as an indicator of subcutaneous fat, accessible and easy to measure through a single skinfold, whose measurement can effectively identify an excess of weight [20], in addition to the deposit of fat that occurs with increasing BMI and age [21]. In people with an ID, some body-composition indicators such as waist circumference, fat area, skinfolds, and waist-to-height ratio have been positively correlated with cardiometabolic risk factors [22]. However, the submandibular skinfold has not been validated and is not usually considered a predictor of cardiometabolic risk in this population, despite its easy and rapid application compared to other more invasive anthropometric measurements that require more instruments and evaluation time. To be considered as a reliable test, the skinfold measurement should have small changes in the mean, a low standard error of measurement, and a high test–retest correlation between repeated trials [23]. To ensure the above, a flexible inelastic tape and skinfold calipers are employed to perform skinfold measurements in the different regions, standardizing the used protocols [24]. In this study, the submandibular fold was measured following the above, by applying a caliper and repeating the same protocol each time. With this, the aim of the present study was to primarily validate the submandibular skinfold as an anthropometric measurement of cardiometabolic risk in children, adolescents, and adults with an ID.

## 2. Materials and Methods

### 2.1. Design and Participants

The present was a descriptive, non-experimental, and cross-sectional study. The sample consisted of 131 people with mild and moderate ID of both sexes (67.2% men), belonging to two special educational centers and two non-schooled social groups of people with ID from the city of Santiago, Chile. The data of the participants was extracted in the context of the development of ¨Ludo-Inclusion 19¨ project, belonging to the Vice-rector’s Office for Community Outreach (VIME, in Spanish) of Universidad de Santiago de Chile, between the months of August and November of 2021. The parents and/or guardians of the participants had to sign an informed consent prior to conducting the study, where they voluntarily approved the participation of their pupils. For its part, the authorization of the authorities belonging to the centers and groups involved was required. The study complies with the guidelines set forth in the Declaration of Helsinki (2014) and has the approval of the ethics committee of the University of Granada, code 2052/CEIH/2021. The following inclusion criteria were considered: (1) diagnosis of mild or moderate ID, intelligence quotient (IQ) (≤69 and ≥49), obtained through the “Wechsler Intelligence Scale for Children” or WISC III [25] in the case of minors (<18 years), and using the WAIS IV or “Wechsler Intelligence Scale for Adults-IV” [26] in the case of participants >18 years, whose information was obtained from the clinical records of each center; and (2) have independent autonomy and mobility. The following exclusion criteria were considered: (1) having some type of motor disability and (2) being dependent on a wheelchair. The groups were classified according to age into three categories (a) children: 5–11 years; (b) adolescents: 12–17 years; (c) adults: 18–45 years. There was no pubertal stage or maturation differentiation between the groups of children and adolescents The groups categorization by age were founded in evidence of different existing percentiles of anthropometric measurements by sex and age, as well as cut-off points to assess central obesity in children, adolescents, and adults. In fact, BMI and waist circumference show differences by age [27,28], tending to increase its values with age [29] and suggesting that mortality risk is also age related [30].

### 2.2. Cardiometabolic Risk

**Figure 1:**
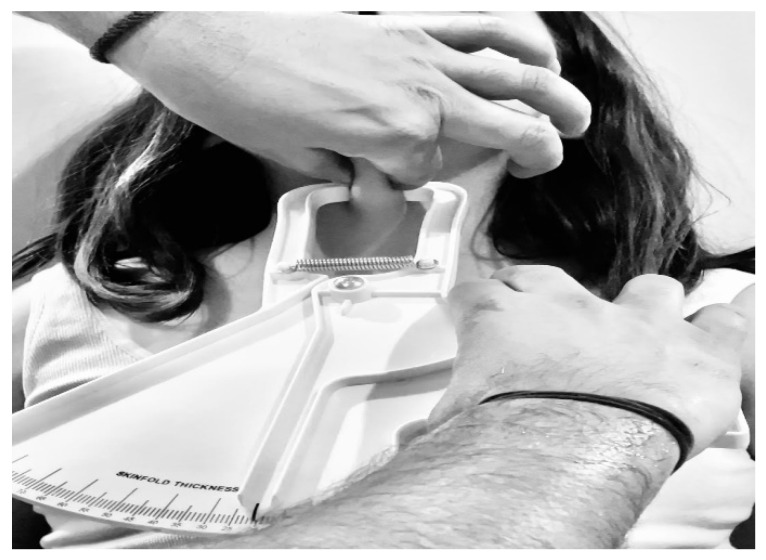
Submandibular fold measurement protocol.

The cardiometabolic risk indicators used in the present study were: body weight (kg), height (cm), BMI (kg/m^2^), neck circumference (cm), submandibular skinfold (cm), waist circumference (cm), calf circumference (cm), and waist-to-height ratio. Body weight and height were measured following the protocol of the World Health Organization [31], with the participants wearing light clothing and without shoes, using a 206 SECA model digital scale and a portable stadiometer, respectively. Neck, abdomen, and calf circumferences were measured using a 201-SECA-model inelastic measure tape. The measurement of the neck circumference was performed standing and in an upright position, with the head positioned in the Frankfort horizontal plane, placing the measure tape at the midpoint of the neck height [32]. Waist circumference was measured at the midpoint between the iliac crest and the last rib, at the end of the expiratory movement [31], while the calf circumference was measured in the widest section of the distance between the ankle and the knee, in the calf area [33]. The measurement of the submandibular fold was made by applying a Slim Guide caliper (Rosscraft, Surrey, Canada) previously validated [34], in a bipedal position and looking forward, at the point of the line that joins the thyroid cartilage and the chin, in an anteroposterior direction [35], as Figure 1 shows. The waist–height ratio (WC (cm)/height (cm)) was calculated based on the absolute values of the aforementioned measurements. The indicators used to quantify obesity were BMI and waist circumference, which have been established as easy-to-apply tools in clinical practice to assess cardiovascular risk in overweight or obese patients [36].

### 2.3. Statistic Analysis

The results were analyzed using version 26 SPSS software (SPSS Inc., IBM Corp., Armonk, New York, NY, USA). The Kolmogorov–Smirnov test was performed to assess the normality distribution of the data. For continuous variables, mean and standard deviation (SD) were presented and for categorical variables, frequency and percentages. Pearson’s I and Spearman’s correlation coefficients for body weight, height, neck circumference, waist circumference, calf circumference, BMI, and waist–height ratio were used. Correlation values I < 0.30 were considered negligible, 0.30–0.49 low, 0.50–0.69 moderate, 0.70–0.89 high and 0.90–1.00 very high [37] and intraclass correlation (ICC) (neck circumference, waist circumference, and calf circumference) to establish concordance between the variables obtained from different anthropometric tests and the submandibular skinfold. ICC values < 0.50 were considered low reliability, values between 0.50 and 0.75 were considered moderate reliability, values between 0.75 and 0.90 were considered good reliability and values between 0.90 and 1.00 were considered excellent reliability [38]. Finally, Bland–Altman plots were made to perform the graphical representation of comparison between submandibular fold and neck, waist, and calf circumference. A significance level of 5% (*p* < 0.05) was considered.

## 3. Results

The sample included a total of 131 people (67.2% men), with a mean age of 16 years (SD: 7.2) among the three age categories. The means for body weight and height were 57.9 (SD: 23.1) and 1.51 (0.17), respectively. The highest BMI was recorded in the adult group, with a value of 27.8 kg/m^2^ (SD: 8.0). The means for neck, waist, and calf circumference were established at 35.0 cm (SD: 5.7), 79.1 cm (SD: 16.8) and 32.8 cm (SD: 5.7), respectively. The largest submandibular skinfold measurement was observed in the group of adults, with an average of 0.89 cm (SD: 0.40) (Table 1).

**Figure 2:**
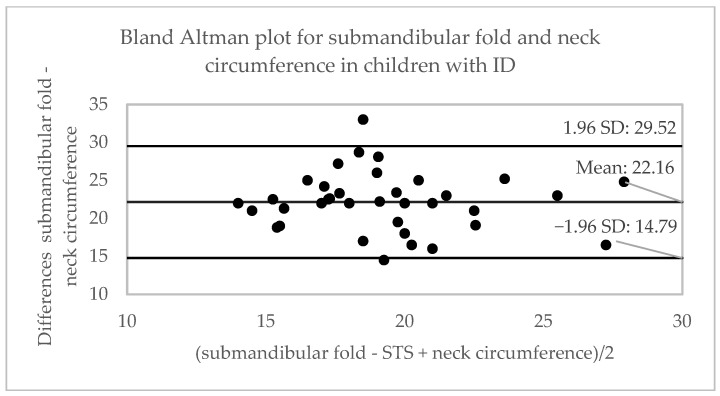

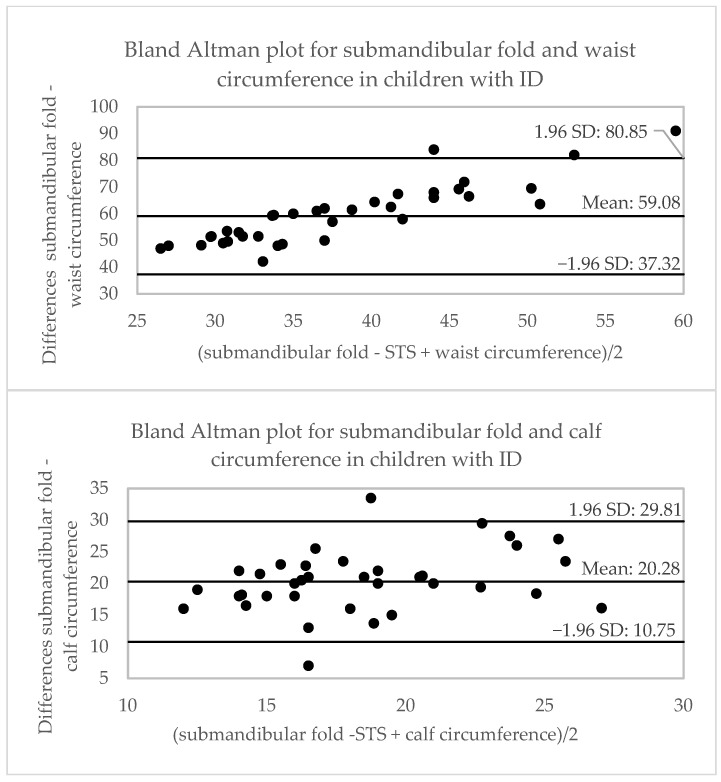
Bland–Altman plots for the comparison between the submandibular skinfold and neck, waist, and calf circumferences in children with ID. STS: spur-to-spur; SD: standard deviation.

**Figure 3:**
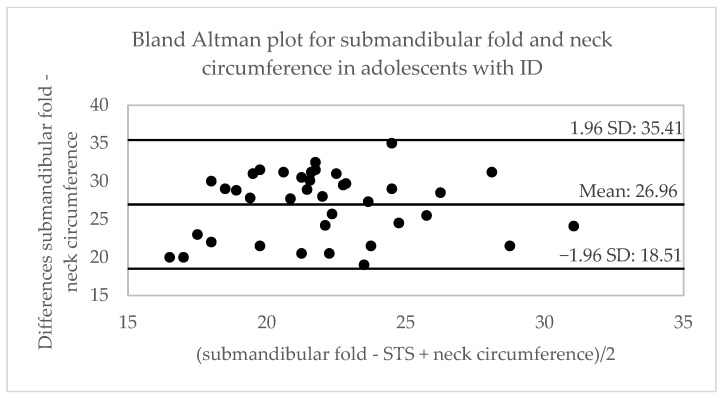

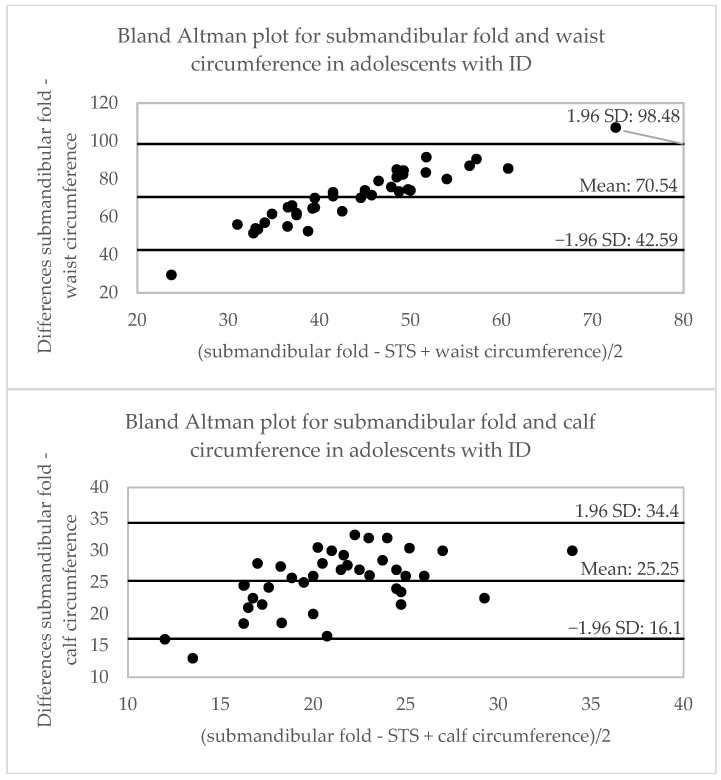
Bland–Altman plots for the comparison between the submandibular skinfold and neck, waist, and calf circumferences in adolescents with DI. STS: spur-to-spur; SD: standard deviation.

**Figure 4:**
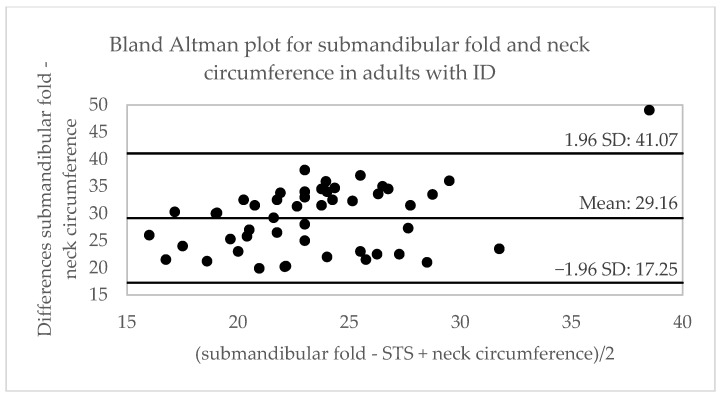

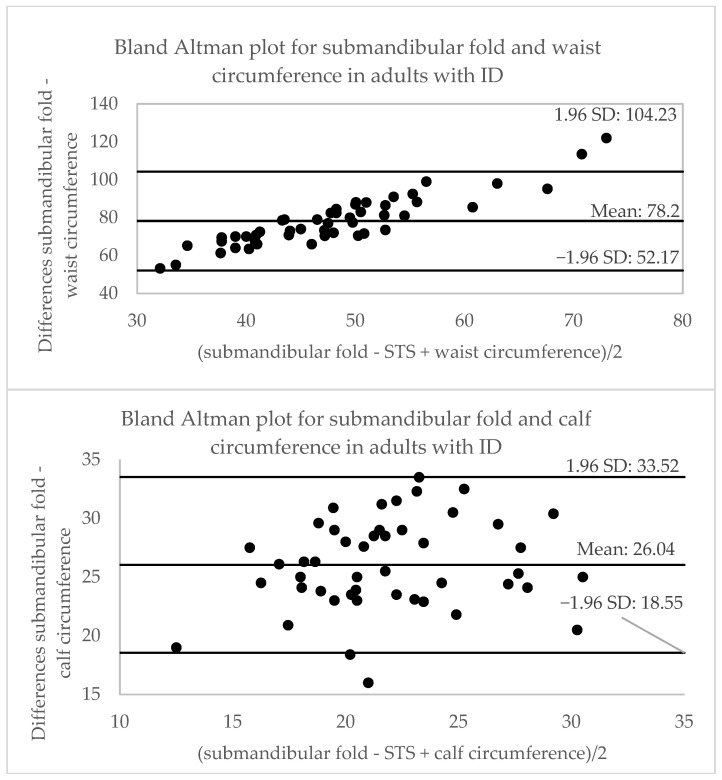
Bland–Altman plots for the comparison between the submandibular skinfold and neck, waist, and calf circumferences in adults with DI. STS: spur-to-spur; SD: standard deviation.

Table 2 shows the correlation between the variables obtained from different anthropometric tests. A moderate correlation is observed between the measurement of the submandibular fold (cm) and body weight (r = 0.48), waist circumference (r = 0.53) and neck-abdomen index (r = 0.53); and a high correlation with BMI (r = 0.63) and waist-to-height ratio (r = 0.62), for the three age categories. The highest correlation between tests (r = 0.70) was established in the adolescent group for BMI and waist–height ratio in adults. The concordance between the tests is observed in Table 2 and Figure 2, Figure 3 and Figure 4, showing a moderate concordance (ICC = 0.66) between the submandibular fold measurement and calf circumference in all groups. In the analysis via age, the highest concordance was established between the submandibular fold test and neck circumference (ICC = 0.67) in children and the calf circumference in adolescents (ICC = 0.71) and adults (ICC = 0.75), respectively.

## 4. Discussion

The present study aimed to primarily validate the submandibular skinfold as an anthropometric measure of cardiometabolic risk in Chilean children, adolescents, and adults with an ID. The main results of the study show a moderate correlation between the submandibular skinfold measurement and the anthropometric measurements analyzed in all age groups, as well as significant correlations between the submandibular skinfold and calf and neck circumferences, in the adolescent/adult and children group, respectively.

The measurement of BMI in the general population has been widely used because, together with total adiposity, it correlates positively with the risk of cardiometabolic disease [39], which in the case of children with an ID exceeds the values recommended by the World Health Organization [40], making them more prone to obesity from an early age. Our study shows a high BMI in the adult group, which coincides with previous studies in people with an ID that show an increase in BMI and body weight in the age groups of 18 to 29 and 30 to 39 years, and then decrease when older [41]. In an ID population, BMI and waist circumference have been established as appropriate measurements, not recommending skinfold thickness measurements due to participant noncompliance and eventual lack of precision and inaccurate results [42]. However, other authors have proposed other anthropometric indicators in addition to BMI and waist circumference, such as skinfolds, hip circumference and relaxed arm circumference, adjusted by height to define metabolic syndrome in a population with ID [43]. Along the same line, the present study proposes the measurement of the submandibular skinfold as a non-invasive anthropometric measurement, showing the highest values in the adult group. In studies of obese children, significant associations between neck circumference and body weight, height, waist circumference, hip circumference, and skinfold thickness were found only in normal-weight girls and not in boys, nor in both sexes with obesity [44]. Other studies propose the submandibular skinfold as a new measure to assess nutritional status and obesity in newborns and children, demonstrating a high correlation between it and BMI, the sum of the four conventional skinfolds, arm circumference, arm fat area, and body fat percentage [34]. Consistent with previous studies, our results demonstrated a moderate correlation between the submandibular skinfold measurement and body weight, waist circumference, and neck–abdomen ratio, as well as a high correlation between BMI and waist-to-height ratio measurements in all age groups. Likewise, the submandibular skinfold has been shown to correlate with BMI, body weight, neck circumference, brachial circumference, and bicep and tricep skinfolds in young adults [45]. In addition, studies to estimate the cardiometabolic risk in people with HIV have established significant correlations between neck circumference and BMI, waist circumference, hip circumference, waist–hip ratio, and waist–height ratio [46]. Skinfold-thickness measurements have been demonstrated to have correlation with other health parameters such as abnormal glucose and insulin regulation [47], while arterial stiffness has been presented as an indicator of hypertension [48]. The submandibular skinfold has specifically been associated with high blood pressure, diabetes mellitus, and inguinal hernias in both sexes [21].

As a result, the submandibular skinfold is presented as an easy and accessible anthropometric measurement, which is related to other anthropometric measurements and body-composition indexes. Therefore, this measure could be included as a minimally invasive and low-cost alternative for the anthropometric evaluation of people with an ID, being part of the physical and nutritional evaluations of this population.

Some limitations of the study in regard to participants was the no-differentiation according to the Tanner scale for the assessment of pubertal stage in children in comparison with the other age groups, as well as the differentiated information for each syndrome associated with intellectual disability. In other aspects, the lack of consideration of the lifestyle-associated variables of the participants such as physical activity level, nutritional status, and associated diseases could have influenced the obtained results. Moreover, the study did not consider gold standard methods, such as magnetic resonance imaging (MRI) or dual X-ray absorptiometry (DXA) for the assessment of body composition, specifically fat mass, lean body mass, bone mass, and/or muscle mass [49]. In this context, the proposed skinfold could be used as a subcutaneous fat measurement to make a comparison between fat mass and cardiovascular risk factors in populations with ID. In addition, no correlation was established by sex or adjustment according to sociodemographic variables or other factors that could give greater value to the results according to the categories. One of the strengths of the study is the novelty it brings in being focused on establishing correlations of the submandibular fold with specific anthropometric measurements for Chilean people with an ID, whose population and country lack similar studies. In addition, the division by age groups makes possible a more segmented analysis of the population with ID, establishing parameters and conclusions for each group according to their anthropometric characteristics and specific needs for their application in school contexts, thus proposing a new alternative for assessing cardiometabolic risk that could be incorporated into future assessment batteries for the physical condition of people with an ID.

## 5. Conclusions

The present study showed moderate correlations between the submandibular skinfold measure and the anthropometric measurements analyzed (body weight, waist circumference, BMI, and waist-to-height ratio) in the three age categories, showing the highest correlation between the submandibular skinfold and BMI in the adolescent group and waist-to-height ratio in adults. As a result, our study suggests a great potential of the submandibular skinfold as a minimally invasive anthropometric measurement, one capable of an easy and rapid estimation in assessing cardiometabolic risk in people with an ID. Further studies should be carried out on this subject, incorporating this skinfold as a body-fat measurement in relation to cardiovascular risk factors in children, adolescents, and adults with an ID and as a method of research, evaluation, and the monitoring of physical and nutritional status in this population.

## Figures and Tables

**Table 1:** Descriptive analysis (n [%] or mean [SD]) of indicators of overweight/obesity by age category.

Variable	Total (n = 131)	Children (n = 43)	Adolescents (n = 39)	Adults (n = 49)	*p* Value
Age (years)—Average (SD)	16 (7.2)	8.2 (2.1)	14.1 (1.5)	23.2 (5.2)	
Sex—n (%)					
Women	43 (32.8)	12 (27.9)	12 (30.8)	19 (38.8)	
Men	88 (67.2)	31 (72.1)	27 (69.2)	30 (61.2)	
Anthropometry—Average (SD)					
Body weight (kg)	57.9 (23.1)	35.3 (15.7)	63.6 (21.4)	69.2 (18.2)	<0.001 ^a^**
Height (cm)	1.51 (0.1)	1.33 (0.1)	1.61 (0.1)	1.58 (0.1)	<0.001 ^a^**
Neck circumference (cm)	35.0 (5.7)	30.0 (3.6)	35.4 (3.9)	38.0 (6.0)	<0.001 ^a^**
Submandibular fold (cm)	0.85 (0.3)	0.80 (0.3)	0.85 (0.3)	0.89 (0.4)	0.628
Waist circumference (cm)	79.1 (16.8)	66.1 (13.6)	79.0 (16.2)	87.1 (15.1)	<0.001 ^b^**
Calf circumference (cm)	32.8 (5.7)	27.9 (5.4)	33.7 (5.9)	34.9 (4.5)	<0.001 ^a^**
BMI (kg/m^2^)	24.2 (6.8)	18.8 (5.8)	24.4 (6.4)	27.8 (8.0)	<0.001 ^a^**
Waist–height ratio	0.51 (0.1)	0.49 (0.1)	0.49 (0.0)	0.55 (0.1)	<0.001 ^b^**

SD: Standard deviation; BMI: body mass index; ^a^ = ANOVA one way; ^b^ = Kruskal–Wallis; significance value ** *p* = < 0.001.

**Table 2:** Correlation between variables obtained from different anthropometric tests by age categories.

Variables	Total (n = 131)		Children (n = 43)		Adolescents (n = 39)		Adults (n = 49)	
	Submandibular Fold (cm)							
	r	ICC	r	ICC	r	ICC	r	ICC
Body weight (kg)	0.48 **		0.57 **		0.56 **		0.51 **	
Height (cm)	0.08	0.01	0.43 **	0.06	−0.01	0.00	−0.34	0.03
Neck circumference (cm)	0.35 **	0.49 **	0.51 **	0.67 **	0.38 *	0.55 **	0.31 *	0.45 *
Waist circumference (cm)	0.53 ^b^**	0.39 **	0.58 ^b^**	0.50 *	0.53 ^b^**	0.42 *	0.56 ^b^**	0.44 *
Calf circumference (cm)	−0.39 **	0.66 **	0.48 **	0.63 **	0.61 **	0.71 **	0.61 **	0.75 **
BMI (kg/m^2^)	0.63 **		0.65 **		0.70 **		0.63 **	
Waist–height ratio	0.62 ^b^**		0.54 ^b^**		0.60 ^b^**		0.70 ^b^**	

* *p* < 0.05; ** *p* = < 0.001. r: Pearson (p); ^b^ Spearman (np); ICC: Intraclass correlation.

## Data Availability

The data that support the findings of this study are available from the corresponding author upon reasonable request.
